# Supplementary material for: Multi‐dataset identification of innovative feature genes and molecular mechanisms in keratoconus
Source: J Cell Mol Med. 2024 Sep 19;28(18):e70079. doi: 10.1111/jcmm.70079 (PMC11412914; doi:10.1111/jcmm.70079)
Supplement: Supplementary file 2 — Material S2. [file JCMM-28-e70079-s002.zip › 7.GSEA analysis/3.DBNDD1/KEGG_RETINOL_METABOLISM.html]

Details for gene set KEGG\_RETINOL\_METABOLISM[GSEA]

|  || Dataset | DBNDD1.group.cls#h\_versus\_l |
| Phenotype | group.cls#h\_versus\_l |
| Upregulated in class | l |
| GeneSet | KEGG\_RETINOL\_METABOLISM |
| Enrichment Score (ES) | -0.37862107 |
| Normalized Enrichment Score (NES) | -1.217362 |
| Nominal p-value | 0.13861386 |
| FDR q-value | 0.7047534 |
| FWER p-Value | 0.987 |
Table: GSEA Results Summary

  

Fig 1: Enrichment plot: KEGG\_RETINOL\_METABOLISM      
 Profile of the Running ES Score & Positions of GeneSet Members on the Rank Ordered List

  

| SYMBOL | TITLE | RANK IN GENE LIST | RANK METRIC SCORE | RUNNING ES | CORE ENRICHMENT || 1 | UGT1A8 | na | 313 | 0.502 | 0.0478 | No |
| 2 | RPE65 | na | 941 | 0.390 | 0.0739 | No |
| 3 | UGT2B11 | na | 1393 | 0.345 | 0.0999 | No |
| 4 | UGT2B4 | na | 2640 | 0.266 | 0.0941 | No |
| 5 | UGT1A6 | na | 2660 | 0.265 | 0.1236 | No |
| 6 | RDH12 | na | 3612 | 0.227 | 0.1218 | No |
| 7 | CYP26C1 | na | 3713 | 0.223 | 0.1442 | No |
| 8 | ADH6 | na | 3734 | 0.222 | 0.1688 | No |
| 9 | CYP2C18 | na | 4789 | 0.187 | 0.1595 | No |
| 10 | ADH1B | na | 5723 | 0.158 | 0.1505 | No |
| 11 | UGT2B28 | na | 6027 | 0.150 | 0.1587 | No |
| 12 | CYP2C9 | na | 7646 | 0.111 | 0.1246 | No |
| 13 | CYP3A4 | na | 8583 | 0.093 | 0.1081 | No |
| 14 | RDH16 | na | 9547 | 0.078 | 0.0891 | No |
| 15 | RETSAT | na | 10061 | 0.071 | 0.0823 | No |
| 16 | CYP2C8 | na | 10615 | 0.063 | 0.0735 | No |
| 17 | DGAT2 | na | 10922 | 0.059 | 0.0713 | No |
| 18 | UGT2A3 | na | 13213 | 0.036 | 0.0094 | No |
| 19 | RDH10 | na | 14014 | 0.030 | -0.0103 | No |
| 20 | CYP2B6 | na | 14096 | 0.029 | -0.0093 | No |
| 21 | CYP2A7 | na | 15639 | 0.018 | -0.0517 | No |
| 22 | ALDH1A2 | na | 16724 | 0.011 | -0.0817 | No |
| 23 | UGT2B10 | na | 17872 | 0.004 | -0.1144 | No |
| 24 | DHRS4 | na | 17878 | 0.004 | -0.1141 | No |
| 25 | ADH7 | na | 17985 | 0.003 | -0.1169 | No |
| 26 | CYP2C19 | na | 18485 | -0.001 | -0.1312 | No |
| 27 | DGAT1 | na | 18959 | -0.004 | -0.1443 | No |
| 28 | UGT2B15 | na | 18962 | -0.004 | -0.1439 | No |
| 29 | CYP2A13 | na | 19140 | -0.006 | -0.1484 | No |
| 30 | PNPLA4 | na | 19750 | -0.011 | -0.1647 | No |
| 31 | ADH5 | na | 20394 | -0.016 | -0.1814 | No |
| 32 | CYP26A1 | na | 20582 | -0.018 | -0.1848 | No |
| 33 | DHRS4L2 | na | 20818 | -0.020 | -0.1892 | No |
| 34 | BCO1 | na | 21532 | -0.027 | -0.2067 | No |
| 35 | ALDH1A1 | na | 21872 | -0.031 | -0.2130 | No |
| 36 | DHRS3 | na | 22848 | -0.041 | -0.2365 | No |
| 37 | RDH11 | na | 23838 | -0.054 | -0.2589 | No |
| 38 | UGT2B7 | na | 24690 | -0.066 | -0.2759 | No |
| 39 | RDH5 | na | 24832 | -0.068 | -0.2722 | No |
| 40 | CYP3A7 | na | 26069 | -0.089 | -0.2978 | No |
| 41 | CYP1A2 | na | 27673 | -0.123 | -0.3302 | No |
| 42 | UGT2A1 | na | 28049 | -0.132 | -0.3260 | No |
| 43 | CYP4A11 | na | 29497 | -0.172 | -0.3483 | No |
| 44 | ADH1A | na | 29956 | -0.187 | -0.3403 | No |
| 45 | LRAT | na | 31286 | -0.232 | -0.3523 | Yes |
| 46 | CYP3A43 | na | 31748 | -0.251 | -0.3372 | Yes |
| 47 | CYP3A5 | na | 31787 | -0.253 | -0.3096 | Yes |
| 48 | ADH4 | na | 32701 | -0.306 | -0.3013 | Yes |
| 49 | CYP4A22 | na | 32907 | -0.321 | -0.2708 | Yes |
| 50 | CYP26B1 | na | 33344 | -0.357 | -0.2430 | Yes |
| 51 | ADH1C | na | 33609 | -0.380 | -0.2076 | Yes |
| 52 | AWAT2 | na | 34088 | -0.442 | -0.1714 | Yes |
| 53 | CYP1A1 | na | 34212 | -0.466 | -0.1222 | Yes |
| 54 | RDH8 | na | 34498 | -0.551 | -0.0681 | Yes |
| 55 | DHRS9 | na | 34636 | -0.660 | 0.0027 | Yes |
Table: GSEA details [plain text format]

  

Fig 2: KEGG\_RETINOL\_METABOLISM      
 Blue-Pink O' Gram in the Space of the Analyzed GeneSet

  

Fig 3: KEGG\_RETINOL\_METABOLISM: Random ES distribution      
 Gene set null distribution of ES for **KEGG\_RETINOL\_METABOLISM**
